# Supplementary material for: Control of Clostridioides difficile virulence and physiology by the flagellin homeostasis checkpoint FliC-FliW-CsrA in the absence of motility
Source: mBio. 2025 Jan 30;16(3):e03801-24. doi: 10.1128/mbio.03801-24 (PMC11898703; doi:10.1128/mbio.03801-24)
Supplement: Supplemental material — Fig. S1-S8 and Tables S1-S3. [file mbio.03801-24-s0001.docx]

**SUPPLEMENTAL MATERIAL**

**Supplementary Figures**


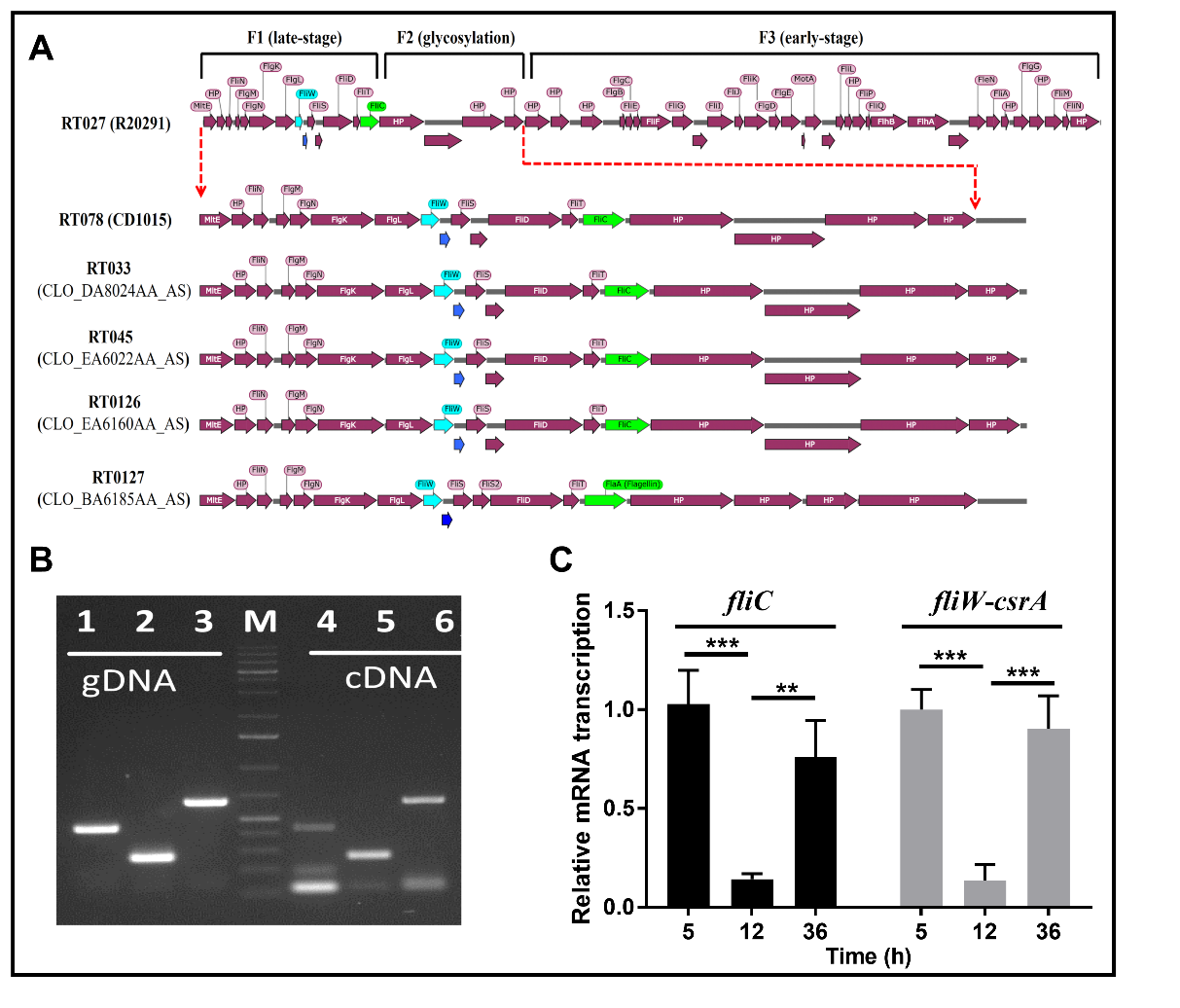


**Fig. S1 Conservation of flagellar genes in non-motile clade 5 strains and verification of *fliW*-*csrA* and *fliC* transcription.**

**(A)** Schematic representation of flagellar genes in the non-motile clade 5 strains and motile RT027 R20291. **(B)** Verification of *fliW* and *csrA* co-transcription by RT-PCR. M: DNA ladder; 1-3: CD1015 genomic DNA was used as PCR template; 4-6: CD1015 cDNA was used as PCR template. 1 and 4: *fliW* PCR test; 2 and 5: *csrA* PCR test; 3 and 6: *fliW*-*csrA* PCR test. **(C)** Transcription analysis of *fliC* and *fliW*-*csrA* in CD1015. Bars stand for mean ± SEM. Differences were considered statistically significant if *P* < 0.05 (***P* < 0.01, ****P* < 0.001). One-way ANOVA with post-hoc Tukey test was used for statistical significance.


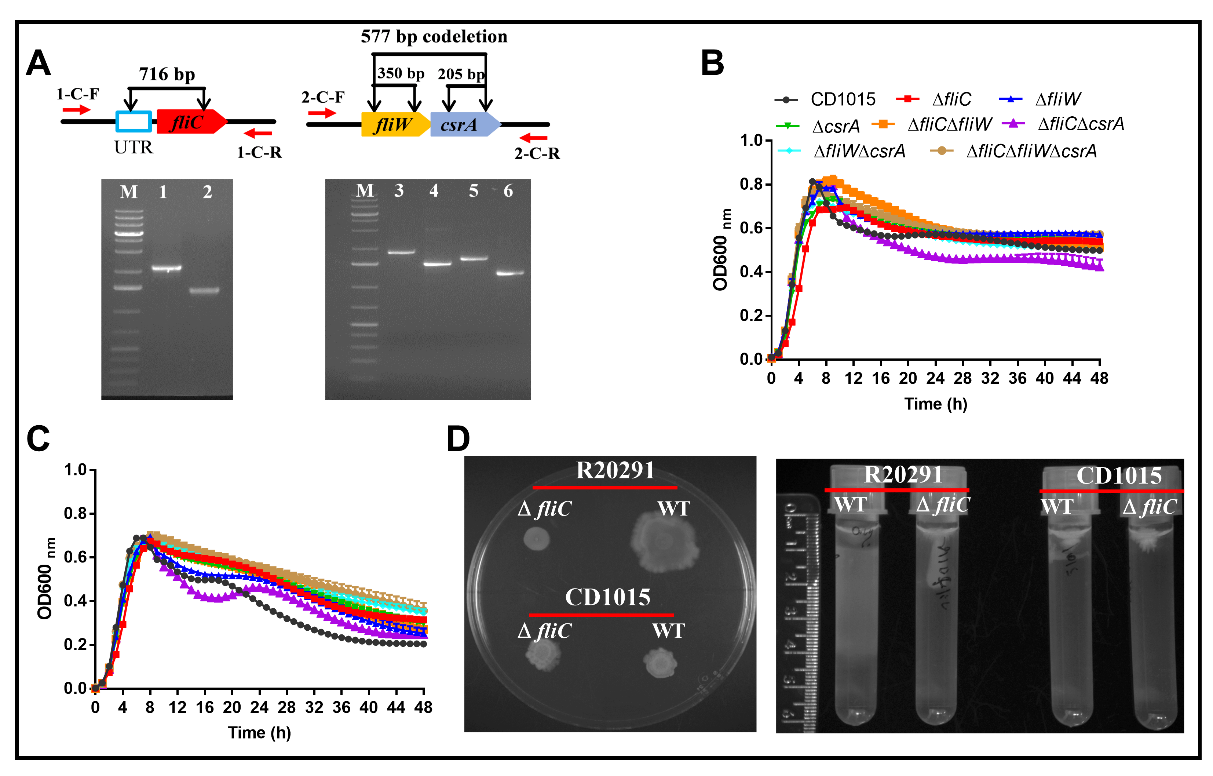


**Fig. S2 Generation of CD1015 derivative mutants, and test of bacterial growth profiles and motility.**

**(A)** Deletion of UTR-*fliC*, *fliW*, *csrA*, and *fliW*-*csrA*. 1-C-F/R were used to verify *fliC* deletion and 2-C-F/R were used to test *fliW*, *csrA*, and *fliW*-*csrA* deletion. M: DNA ladder; 1 and 3: CD1015 genome as PCR template; 2: CD1015∆*fliC* (∆*fliC*) genome test; 4: CD1015∆*fliW* (∆*fliW*) genome test; 5: CD1015∆*csrA* (∆*csrA*) genome test; 6: CD1015∆*fliW*∆*csrA* (∆*fliW*∆*csrA*) genome test. **(B)** Growth profile in BHIS media. **(C)** Growth profile in TY media. **(D)** Bacterial motility test. Swarming and swimming analysis were tested with soft BHIS agar (0.2%) plates and BHIS agar (0.15%) tubes, respectively.


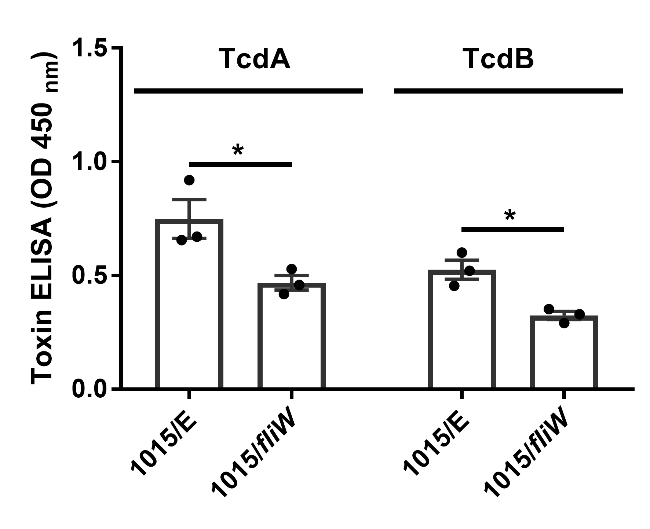


**Fig. S3. Toxin expression in *fliW* overexpression strain.**

TcdA and TcdB concentrations in the supernatants of CD1015-pMTL84153 (1015/E) and *fliW* overexpression strain (1015/*fliW*) were detected by ELISA. Bars stand for mean ± SEM. Differences were considered statistically significant if *P* < 0.05 (**P* < 0.05). Statistical analysis was performed using an unpaired two-tailed t-test.


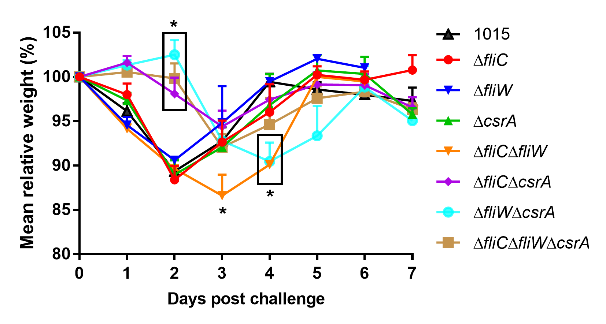


**Fig. S4 Weight loss of CD1015 and its derivative mutants in a CDI mouse model.**

Mouse weight was measured daily for 7 days. Notably, only 1-3 mice survived in some mutant infection groups beyond 3 days post-infection. Statistical significance was determined at *P* < 0.05 (**P* < 0.01), using a paired t-test.


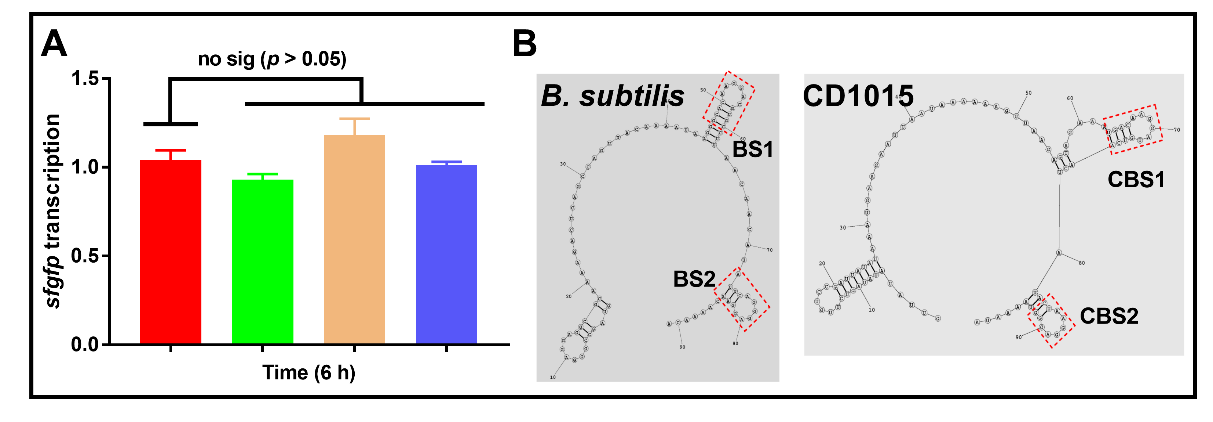


**Fig. S5 Characterization of FliC-FliW-CsrA regulation loop in *C. difficile.***

**(A)** Transcription of *sfgfp* in the different recombinant reporter strains. Red column: P*_lacZ_*-UTR-*sfgfp*; Green column: P*_lacZ_*-UTR-*sfgfp*-P*_tet_*-*fliW*; Orange column: P*_lacZ_*-UTR-*sfgfp*-P*_tet_*-*csrA*; Blue column: P*_lacZ_*-UTR-*sfgfp*-P*_tet_*-*fliW*-*csrA*; **(B)** Structure comparison of *B. subtilis* 5’-UTR of *hag* and CD1015 5’-UTR of *fliC*. BS1 and BS2: CsrA binding sites 1 and 2 in *B. subtilis*. CBS1 and CBS2: CsrA potential binding sites 1 and 2 in CD1015. RNAstructure [dynalign](https://rna.urmc.rochester.edu/RNAstructureWeb/Servers/dynalign/dynalign.html) results were calculated (calculate the lowest free energy secondary structures common to two unaligned sequences).


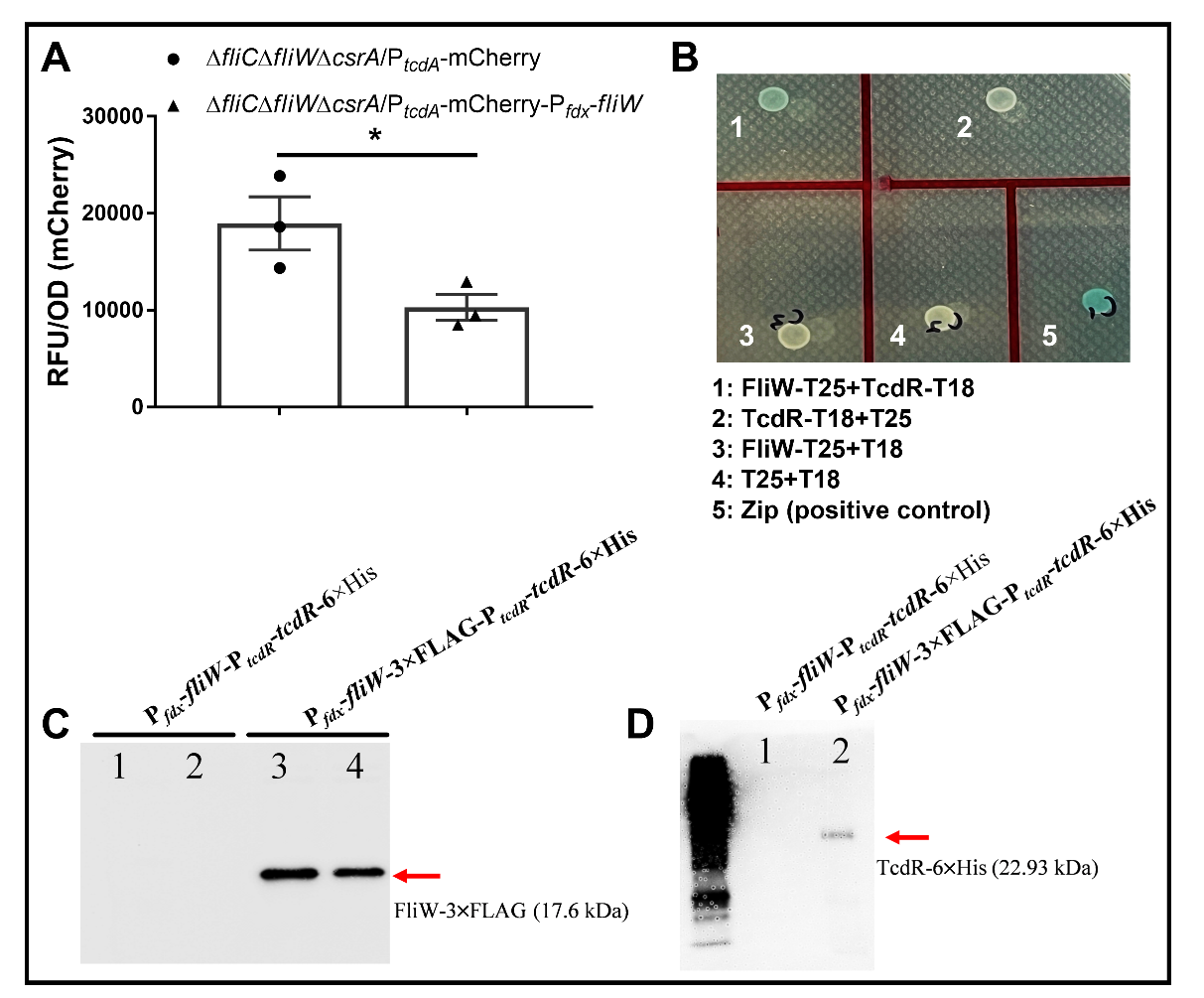


**Fig. S6 FliW interacts with the toxin expression positive regulator TcdR.**

**(A)** Expression of an mCherry reporter driven by the *tcdA* promoter with or without *fliW* co-expression in the Δ*fliC*Δ*fliW*Δ*csrA* mutant. Bacterial pellets were collected from 48 h post incubation and washed three times by PBS for fluorescence detection. The relative fluorescence unit was normalized to the bacterial OD_600nm_. Statistical significance was determined at *P* < 0.05 (**P* < 0.05), using a paired t-test. **(B)** Protein-protein interaction test between FliW and TcdR *in vitro* using a bacterial two-hybrid system*.* **(C)** Verification of FliW-3×FLAG expression in the Δ*fliC*Δ*fliW*Δ*csrA*Δ*tcdR* mutant with anti-FLAG antibody by Western blotting. Cell lysis from 36 h post-incubation bacteria in TY media were used for detection. **(D)** Detection of TcdR-6×His by Western blotting following co-immunoprecipitation (Co-IP). FliW-3×FLAG served as the bait protein to capture TcdR-6×His and FliW lacking the 3×FLAG tag was used as a control. All constructs were conducted in the Δ*fliC*Δ*fliW*Δ*csrA*Δ*tcdR* mutant.


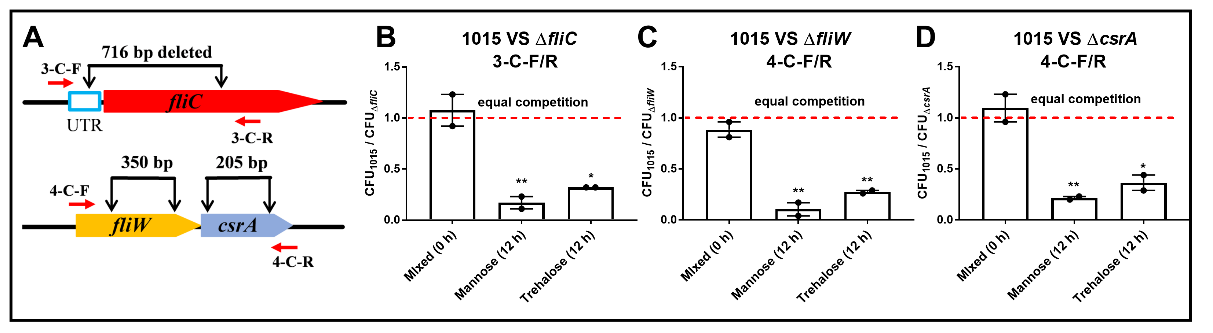


**Fig. S7 ∆*fliC*, ∆*fliW*, and ∆*csrA* mutants outcompete CD1015 in the presence of trehalose and mannose.**

**(A)** Primers used for the colony PCR in the competition test. **(B-D)** CFU_1015_/CFU_mutant_ in the competition cultures. * means the significant difference of CD1015 CFU in the competition mixtures compared to that of at 0 h (**P* < 0.05, ***P* < 0.01). One-way ANOVA with post-hoc Tukey test was used for statistical significance.


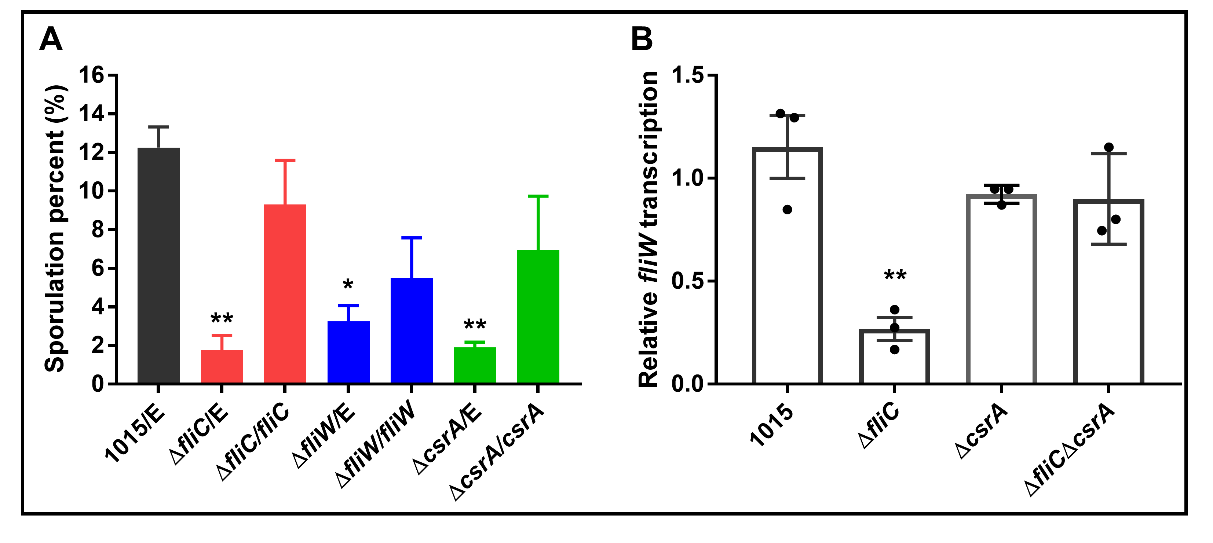


**Fig. S8 Analysis of sporulation and *fliW* transcription in CD1015 and its derivative mutants.**

**(A)** Sporulation analysis. *C. difficile* strains were cultured on 70:30 sporulation agar plates for 5 days, following which the scraped cultures were 10-fold diluted and plated on BHIS plates with 0.1% TA to detect sporulation ratio. The sporulation ratio was calculated as CFU (65 ℃ heated, 30 min) / CFU (not heated). **(B)** Comparison of *fliW* transcription in CD1015, ∆*fliC*, ∆*csrA*, and ∆*fliC*∆*csrA*. Bars stand for mean ± SEM. * means the significant difference of experimental strain compared to CD1015/E or CD1015 (**P* < 0.05, ***P* < 0.01). One-way ANOVA with post-hoc Tukey test was used for statistical significance.

**Supplementary Tables**

**Table S1. Function of late-stage (F1) flagellar genes**

| **Protein** | **Function** | **Reference** |
| --- | --- | --- |
| FliN | part of motor switch | (1) |
| FlgM | inhibitor of FliA regulating late gene expression | (1) |
| FlgN | part of cytoplasmic chaperone | (1) |
| FliS1 | part of cytoplasmic chaperone | (1) |
| FliS2 | part of cytoplasmic chaperone | (1) |
| FliT | part of cytoplasmic chaperone | (1) |
| FlgK | hook-filament Junction | (1) |
| FlgL | hook-filament Junction | (1) |
| FliW | regulating bacterial motility and other physiology phenotypes | (2-4) |
| CsrA | carbon storage regulator | (2-4) |
| FliD | filament cap | (1) |
| FliC | filament protein | (1) |

**Table S2. Bacteria and plasmids utilized in this study**

| **Strains or plasmids** | **Genotype** | **Reference** |
| --- | --- | --- |
| **Strains** |  |  |
| *E. coli* DH5α | Cloning host | NEB |
| *E. coli* BL21 | Protein expression host | NEB |
| *E. coli* BTH101 | Protein-protein interaction analysis host | (5) |
| *E. coli* HB101/pRK24 | Conjugation donor | (6) |
| *C. difficile* 1015 | Clinical isolate; ribotype 078 | (7) |
| *C. difficile* R20291 | Clinical isolate; ribotype 027 | (8) |
| 1015/E | 1015 containing blank plasmid pMTL84153 | This work |
| 1015/*fliW* | 1015 containing pMTL84153-*fliW* | This work |
| R20291Δ*fliC* | R20291 deleted *fliC* gene | This work |
| Δ*fliC* | CD1015 deleted *fliC* gene | This work |
| Δ*fliC/*E | Δ*fliC* containing pMTL84153 | This work |
| Δ*fliC/fliC* | Δ*fliC* containing pMTL84153-*fliC* | This work |
| Δ*fliW* | CD1015 deleted *fliW* gene | This work |
| Δ*fliW/*E | Δ*fliW* containing pMTL84153 | This work |
| Δ*fliW/fliW* | Δ*fliW* containing pMTL84153-*fliW* | This work |
| Δ*csrA* | CD1015 deleted *csrA* gene | This work |
| Δ*csrA/*E | Δ*csrA* containing pMTL84153 | This work |
| Δ*csrA/csrA* | Δ*csrA* containing pMTL84153-*csrA* | This work |
| Δ*fliC*Δ*fliW* | CD1015 deleted *fliC* and *fliW* gene | This work |
| Δ*fliC*Δ*fliW/*E | Δ*fliC*Δ*fliW* containing pMTL84153 | This work |
| Δ*fliC*Δ*fliW/fliW* | Δ*fliC*Δ*fliW* containing pMTL84153-*fliW* | This work |
| Δ*fliC*Δ*csrA* | CD1015 deleted *fliC* and *csrA* gene | This work |
| Δ*fliW*Δ*csrA* | CD1015 deleted *fliW* and *csrA* gene | This work |
| Δ*fliW*Δ*csrA/*E | Δ*fliW*Δ*csrA* containing pMTL84153 | This work |
| Δ*fliW*Δ*csrA/fliW* | Δ*fliW*Δ*csrA* containing pMTL84153-*fliW* | This work |
| Δ*fliC*Δ*fliW*Δ*csrA* | CD1015 deleted *fliC*, *fliW*, and *csrA* gene | This work |
| Δ*fliC*Δ*fliW*Δ*csrA/*E | Δ*fliC*Δ*fliW*Δ*csrA* containing pMTL84153 | This work |
| Δ*fliC*Δ*fliW*Δ*csrA/fliW* | Δ*fliC*Δ*fliW*Δ*csrA* containing pMTL84153-*fliW* | This work |
| **Plasmids** |  |  |
| pKNT25 | T25 reporter at C-terminal | (5) |
| pUT18 | T18 reporter at C-terminal | (5) |
| pKT25 | T25 reporter at N-terminal | (5) |
| pUT18C | T18 reporter at N-terminal | (5) |
| pKNT25-FliW (FliW-T25) | *fliW* fused into N-terminal of T25 reporter | This work |
| pKNT25-FliC (FliW-T25) | *fliC* fused into N-terminal of T25 reporter | This work |
| pKNT25-CrA (CsrA-T25) | *csrA* fused into N-terminal of T25 reporter | This work |
| pUT18-FliW(FliW-T18) | *fliW* fused into N-terminal of T18 reporter | This work |
| pUT18-FliC (FliC-T18) | *fliC* fused into N-terminal of T18 reporter | This work |
| pUT18-CsrA (CsrA-T18) | *csrA* fused into N-terminal of T18 reporter | This work |
| pUT18-TcdR (TcdR-T18) | *tcdR* fused into N-terminal of T18 reporter | This work |
| pET21b | Protein expression plasmid | NEB |
| pET21b-UTR-*sfgfp*-P*_tet_*-*csrA* | Reporter part 5’UTR-*sfgfp* and regulation part P_tet_-*csrA* assembled into pET21b | This work |
| pET21b-UTR-*sfgfp*-P*_tet_*-*fliW* | Reporter part 5’UTR-*sfgfp* and regulation part P*_tet_*-*fliW* assembled into pET21b | This work |
| pET21b-UTR-*sfgfp*-P*_tet_*-*fliW*-*csrA* | Reporter part 5’UTR-*sfgfp* and regulation part P*_tet_*- *fliW*-*csrA* assembled into pET21b | This work |
| pET21b-UTR_CBS1_-*sfgfp*-P*_tet_*-*csrA* | Only kept potential binding site 1 in UTR | This work |
| pET21b-UTR_CBS2_-*sfgfp*-P*_tet_*-*csrA* | Only kept potential binding site 2 in UTR | This work |
| pET21b-UTR_DD_-*sfgfp*-P*_tet_*-*csrA* | Two potential binding sites were deleted | This work |
| pMTL84153 | Complementation plasmid | (9) |
| pMTL84153-*fliC* | pMTL84153 containing *fliC* genes | This work |
| pMTL84153-*fliW* | pMTL84153 containing *fliW* gene | This work |
| pMTL84153-*csrA* | pMTL84153 containing *crsA* gene | This work |
| pMTL84153-*fliW*-*csrA* | pMTL84153 containing *fliW* and *crsA* gene | This work |
| pMTL84151-P*_tcdA_*-mCherry | mCherry reporter driven by the promoter *tcdA* | This work |
| pMTL84153-*fliW*-P*_tcdA_*-mCherry | mCherry reporter coexpression with *fliW* | This work |
| pMTL84151-P*_tcdR_*-*tcdR* | *tcdR* expression driven by the *tcdR* promoter | This work |
| pMTL84151-P*_tcdR_*-*tcdR*-6×His | *tcdR* expression with 6×His tag at C-terminal | This work |
| pMTL84153-*fliW*-P*_tcdR_*-*tcdR*-6×His | Coexpression of FliW and TcdR-6×His | This work |
| pMTL84153-*fliW*-3×FLAG-P*_tcdR_*-*tcdR*-6×His | Coexpression of FliW×FLAG and TcdR-6×His | This work |
| pDL1 | AsCpfI based gene deletion plasmid | (10) |
| pUC57-PsRNA | sRNA promoter template | This work |
| pDL1-*fliC* | *fliC* gene deletion plasmid | This work |
| pDL1-*fliW* | *fliW* gene deletion plasmid | This work |
| pDL1-*csrA* | *csrA* gene deletion plasmid | This work |
| pDL1-*fliW-csrA* | *fliW-csrA* gene deletion plasmid | This work |

**Table S3. Primers utilized in this study**

| **Primer** | **Sequence (5’ to 3’)** |
| --- | --- |
| 1-F | AGAGATACAGATGTTGCTTCA |
| 1-R | TCCTTGTGGTTGCTGATTA |
| 2-F | AGATGAAGCAGTACTTATAGGA |
| 2-R | GCTTATATTGTTAGGTGCTGATA |
| 3-F | ATGATGAAGGTTACATTAAAAAAAG |
| 3-R | CTAGCATCCACTATCACCTCTC |
| 4-F | ATGCTAGTAATTTCAAGAAAAAAAG |
| 4-R | TTATTTTAATGACTTTAAAATATTT |
| 5-F | GCATGCCTGCAGGTCGACTCTAGAGATGAGAGTTAATACAAATGTAAGTGCT |
| 5-R | AATTCGAGCTCGGTACCCGGGGATCTCCTAATAATTGTAAAACTCCTTGT |
| 6-F | ACGCCACTGCAGGTCGACTCTAGAGATGAGAGTTAATACAAATGTAAGTGCT |
| 6-R | AATTCGAGCTCGGTACCCGGGGATCTTATCCTAATAATTGTAAAACTCCTTGT |
| 7-F | GCATGCCTGCAGGTCGACTCTAGAGATGATGAAGGTTACATTAAAAAAAG |
| 7-R | AATTCGAGCTCGGTACCCGGGGATCGCATCCACTATCACCTCTCAAT |
| 8-F | GCGGGCTGCAGGGTCGACTCTAGAGATGATGAAGGTTACATTAAAAAAAG |
| 8-R | TTAGTTACTTAGGTACCCGGGGATCTTAGCATCCACTATCACCTCTCAAT |
| 9-F | GCATGCCTGCAGGTCGACTCTAGAGATGCTAGTAATTTCAAGAAAAAAA |
| 9-R | AATTCGAGCTCGGTACCCGGGGATCTTTTAATGACTTTAAAATATTTAT |
| 10-F | ACGCCACTGCAGGTCGACTCTAGAGATGCTAGTAATTTCAAGAAAAAAA |
| 10-R | AATTCGAGCTCGGTACCCGGGGATCTTATTTTAATGACTTTAAAATATTTAT |
| 11-F | TTTAACTTTAAGAAGGAGATATACATATGCGTAAAGGCGAAGAGC |
| 11-R | TGTCGACGGAGCTCGAATTCGGATCCTTAGTGGTGATGGTGATGGTGTTTGTACAGTTCATCCATACCA |
| 12-F | CTTTAAGAAGGAGATATACATCTTGTCCGATTATATAAATGAAGAATTAATAAAAAAGTTAAGAGTAGAAATGACAAGGATGTCAACTATACTAAGGAGGGTAAAATA |
| 12-R | CCTTCTTAAAGTTAAACAAATATTTTACCCTCCTTAGTATAGTTGACATCCTTGTCATTTCTACTCTTAACTTTTTTATTAATTCTTCATTTATATAATCGGACAAGA |
| 13-F | CTTTAAGAAGGAGATATACATCTTGTCCGATTATATAAATGAAGAATTAATAAAAAAGTTAAGAGTAGAAA CAACTATACTAAGGAGGGTAAAATA |
| 13-R | CCTTCTTAAAGTTAAACAAATATTTTACCCTCCTTAGTATAGTTGTTTCTACTCTTAACTTTTTTATTAATTCTTCATTTATATAATCGGACAAGA |
| 14-F | CTTTAAGAAGGAGATATACATCTTGTCCGATTATATAAATGAAGAATTAATAAAAAAGTTAAGAGTAGAAATGACAAGGATGTCAACTATATAAAATA |
| 14-R | CCTTCTTAAAGTTAAACAAATATTTTATATAGTTGACATCCTTGTCATTTCTACTCTTAACTTTTTTATTAATTCTTCATTTATATAATCGGACAAGA |
| 15-F | CTTTAAGAAGGAGATATACATCTTGTCCGATTATATAAATGAAGAATTAATAAAAAAGTTAAGAGTAGAAACAACTATATAAAATA |
| 15-R | CCTTCTTAAAGTTAAACAAATATTTTATATAGTTGTTTCTACTCTTAACTTTTTTATTAATTCTTCATTTATATAATCGGACAAGA |
| 16-F | TGGACAGCAAATGGGTCGGGATCCGCATAAAAATAAGAAGCCTGCATTT |
| 16-R | AAAAACCTCCTTTACTGCAGGAGCTCAGATCTGTTAACG |
| 17-F | AGCTCCTGCAGTAAAGGAGGTTTTTATGATGAAGGTTACATTAAAAAAAGG |
| 17-R | CAGTGGTGGTGGTGGTGGTGCTCGATTACTAGCATCCATTATCACC |
| 18-F | AGCTCCTGCAGTAAAGGAGGTTTTTATGCTAGTAATTTCAAGAAAAAAAGATGAAGC |
| 18-R | CAGTGGTGGTGGTGGTGGTGCTCGATTATTTTAATGACTTTAAAATTTTTAT |
| 19-F | AGCTCCTGCAGTAAAGGAGGTTTTTATGATGAAGGTTACATTAAAAAAAGG |
| 19-R | CAGTGGTGGTGGTGGTGGTGCTCGATTATTTTAATGACTTTAAAATTTTTAT |
| 20-F | TGTGTTACATATGACCATGATTACGATGAGAGTTAATACAAATGTAAGTG |
| 20-R | CGCGTGACGTCGACTCTAGAGGATCTTATCCTAATAATTGTAAAACTCCT |
| 21-F | TGTGTTACATATGACCATGATTACGATGATGAAGGTTACATTAAAAAAAG |
| 21-R | CGCGTGACGTCGACTCTAGAGGATCCTAGCATCCACTATCACCTCTC |
| 22-F | TGTGTTACATATGACCATGATTACGATGCTAGTAATTTCAAGAAAAAAAG |
| 22-R | CGCGTGACGTCGACTCTAGAGGATCTTATTTTAATGACTTTAAAATATTT |
| 23-F | AAAGTTAAAAGAAGAAAATAGAAATATAATCTTTAATTTGAAAAGATTTA |
| 24-R | GATAGCTGATGAGTTGTTACAATATCTACAAGAGTAGAAATTAGTATCTAGTTTAGATGTAGCTCTATCTACAAGAGTAGAAATTAATGGT |
| 25-F | ATATTGTAACAACTCATCAGCTATCTAATTTCTACTCTTGTAGATGTATAGAAAAAACTGCTTCTGC |
| 25-R | ATTAATTCTTCATTTATATAATCGG |
| 26-F | CCGATTATATAAATGAAGAATTAATATTAGGGGCACAACAAAATAGA |
| 26-R | CATGCTGATCTAGATTTCTCCATAGATCAATCAGTATTTCGTCTGCAT |
| 1-C-F | TCACTAGCAGGCTATTCTTCG |
| 1-C-R | TAAAAAGTCTAAGCACTGAACAAT |
| 27-R | GATAGCTGATGAGTTGTTACAATATCTACAAGAGTAGAAATTATCCATCAAGTAATTTCTTACCATATCTACAAGAGTAGAAATTAATGGT |
| 28-R | CATGCTGATCTAGATTTCTCCATAGGAATCAATTAGTATTTCATCTGCAT |
| 29-R | TATACCTTCTTCTTCAGTAGAATATCTACAAGAGTAGAAATTACGTCTGACCAAGAGTAATTATATATCTACAAGAGTAGAAATTAATGGT |
| 30-R | GTACTGCTTCATCTTTTTTTCTTATCTACAAGAGTAGAAATTAAAGATGAAGCAGTACTTATAGGAATCTACAAGAGTAGAAATTAATGGT |
| 31-F | ATATTCTACTGAAGAAGAAGGTATATAATTTCTACTCTTGTAGATTGCAAATGCTTTAAATGCAACA |
| 31-R | ATGTAACCTTCATCATATCTGCG |
| 32-F | AACGCAGATATGATGAAGGTTACATTTATTGAGAGGTGATAGTGGAT |
| 32-R | CATGCTGATCTAGATTTCTCCATAGCTATTACAGGCAACACATTATCTAT |
| 33-F | AACGCAGATATGATGAAGGTTACATAAATAATAAGCTTGGAGGAAGT |
| 33-R | CATGCTGATCTAGATTTCTCCATAGTTAAAAGAGCATCTTTGGATA |
| 34-F | ATAAGAAAAAAAGATGAAGCAGTACTAATTTCTACTCTTGTAGATCAGTTAAAGGAGATTCTGTCACAAA |
| 34-R | ATCCACTATCACCTCTCAATAATGG |
| 35-F | CCATTATTGAGAGGTGATAGTGGATAAATAATAAGCTTGGAGGAAGT |
| 35-R | CATGCTGATCTAGATTTCTCCATAGTTAAAAGAGCATCTTTGGATA |
| 2-C-F | CTTAGTAATGAAACCAATGAAAAAA |
| 2-C-R | CTGCATCTTGGTCTAATGTAGTCAT |
| 36-F | GCAAGTTGAGCGATTTACTTCGGT |
| 36-R | GTACTGGCTCACCTTTGATATT |
| 37-F | GCGGAAATGGTAGAAATG |
| 37-R | ATCAGGTGCTATCAATACTT |
| 38-F | CTTACACCATCTATGATAGTTG |
| 38-R | AAGTTCAAGTTTATGCTCAAT |
| 39-F | CCAAGTGGTACTATACATGCTAT |
| 39-R | TGAGGAACATTCGTAACATCTAT |
| 40-F | AAGAGATAACGGATATGATATAGA |
| 40-R | GTGTTCAGTAGATGTATGGT |
| 41-F | ACCAACCACGCATAATATCTA |
| 41-R | AGTCATACCTATTTCTTCACCTT |
| 42-F | GCTGTCTTCTGGTGTTAG |
| 42-R | TTCTTCCTGCTTGGTCTA |
| 43-F | CTGAAGAAGAAGGTATAGGAT |
| 43-R | CGTCTGACCAAGAGTAAT |
| 44-F | TACGAATTCGAGCTCGGTACCCGGGGATGAAGGACAAAATGATAGAGA |
| 44-R | TATCTTCTTCTCCTTTAGATACCATAAAAACCTCCTAGTATTATTATTT |
| 45-F | ATGGTATCTAAAGGAGAAGAAGATA |
| 45-R | GTAAAACGACGGCCAGTGCCAAGCT TTATTTATATAATTCATCCATACCT |
| 46-F | CTGTATCCATATGACCATGATTACGCTAGCATCCACTATCACCTCTCA |
| 46-R | TTTTGTCCTTCATCCCCGGGTACCGCGTGTAGTAGCCTGTGAAATAAGTA |
| 47-F | TGTGTTACATATGACCATGATTACG ATGATGAAGGTTACATTAAAAAAAG |
| 47-R | CGCGTGACGTCGACTCTAGAGGATCCTACTTGTCATCGTCATCCTTGTAGTCGATGTCATGATCTTTATAATCACCGTCATGGTCTTTGTAGTCGCATCCACTATCACCTCTCAATAAT |
| 48-F | TCGACGTCACGCGTCCATGGAGATC ATTCTACTGCGGTAGTGTGTTA |
| 48-R | GTAAAACGACGGCCAGTGCCAAGCT |
| 3-C-F | AGCTTACTAAACAAGTGAACACAAT |
| 3-C-R | TTATTCCAATTATGAAAATACACTT |
| 4-C-F | GTTCAATTAAAGGCAGCAGAGT |
| 4-C-R | TCCTGAACTCTTACAAGCTCTCTAT |

**References**

1. Stevenson E, Minton NP, Kuehne SA. 2015. The role of flagella in Clostridium difficile pathogenicity. Trends Microbiol 23:275-82.

2. Mukherjee S, Yakhnin H, Kysela D, Sokoloski J, Babitzke P, Kearns DB. 2011. CsrA-FliW interaction governs flagellin homeostasis and a checkpoint on flagellar morphogenesis in Bacillus subtilis. Mol Microbiol 82:447-61.

3. Mukherjee S, Oshiro RT, Yakhnin H, Babitzke P, Kearns DB. 2016. FliW antagonizes CsrA RNA binding by a noncompetitive allosteric mechanism. Proc Natl Acad Sci U S A 113:9870-5.

4. Oshiro RT, Rajendren S, Hundley HA, Kearns DB. 2019. Robust Stoichiometry of FliW-CsrA Governs Flagellin Homeostasis and Cytoplasmic Organization in Bacillus subtilis. mBio 10.

5. Battesti A, Bouveret E. 2012. The bacterial two-hybrid system based on adenylate cyclase reconstitution in Escherichia coli. Methods 58:325-34.

6. Williams DR, Young DI, Young M. 1990. Conjugative plasmid transfer from Escherichia coli to Clostridium acetobutylicum. J Gen Microbiol 136:819-26.

7. Collins J, Robinson C, Danhof H, Knetsch CW, van Leeuwen HC, Lawley TD, Auchtung JM, Britton RA. 2018. Dietary trehalose enhances virulence of epidemic Clostridium difficile. Nature 553:291-294.

8. Stabler RA, He M, Dawson L, Martin M, Valiente E, Corton C, Lawley TD, Sebaihia M, Quail MA, Rose G, Gerding DN, Gibert M, Popoff MR, Parkhill J, Dougan G, Wren BW. 2009. Comparative genome and phenotypic analysis of Clostridium difficile 027 strains provides insight into the evolution of a hypervirulent bacterium. Genome Biol 10:R102.

9. Heap JT, Pennington OJ, Cartman ST, Minton NP. 2009. A modular system for Clostridium shuttle plasmids. J Microbiol Methods 78:79-85.

10. Zhu D, Patabendige H, Tomlinson BR, Wang S, Hussain S, Flores D, He Y, Shaw LN, Sun X. 2021. Cwl0971, a novel peptidoglycan hydrolase, plays pleiotropic roles in Clostridioides difficile R20291. Environ Microbiol doi:10.1111/1462-2920.15529.
